# The Effect of Tyre Size

**Published:** 1909-02-27

**Authors:** 


### Motoring Notes.

### THE EFFECT OF TYRE SIZE.

Now that the pneumatic tyre has had a very considerable trial on motor cars, the correct size and design for various purposes is at last being realised. There is no doubt that hitherto cars have been mainly fitted with tyres of a totally inadequate size in comparison with their horse-power and weight, and it is to this that the excessive cost of tyre upkeep must be attributed. It is obvious that apart from other things the size of the tyre should depend upon the weight it has to carry, and if the engine power is high it is advisable to increase the size somewhat. Of course the size of tyres one can fit on a car depends on the rims, which in many instances, prohibit much variation. The only alternative in this case is removal of the old and the fitting of new rims of a larger size. This is not such a very expensive proceeding as many people imagine, and even if it were the change is cheaper in the end, as, for instance, when a tyre is just too small for the work it has to do. This entails not only frequent trouble in the form of cuts and punctures, but also rapid wear and deterioration of both the cover and inner tube, resulting in frequent renewals. The use of a tyre of a size proportionate to the weight and horse-power of a car will to a great extent obviate all this, in addition to improving the comfort of the passengers, since the increased bulk of air cushion tends to much smoother running of the car. A few years ago I had a small 6-h.p. car which was fitted when I purchased it with 700 by 85 mm. tyres. After one season's wear the treads of these covers were practically cut all over, and in addition I experienced my usual share of punctures. Now, I believe, it is generally understood that the above size is more or less ample for a two-seated car of low horse-power; in fact up to quite recently similar sized cars were fitted as a rule with 65 mm. tyres. Having always had strong prejudices against the small tyre I determined to fit the largest section my rims would take, and so purchased a set of 700 by 90 mm. tyres of the same make as those I discarded. The increase in size was not great, yet it proved very material. At the end of a season's use the covers were unscratched; I had never had a moment's worry with punctures or any other variety of tyre trouble; and the comfort of the car was very considerably enhanced by its much smoother running qualities. The only possible drawback to large section tyres is a somewhat more pronounced tendency to skid on greasy roads, yet in spite of this I have personally no hesitation in saying that the 90 mm. section is the smallest size that should be fitted even on a two-seated car if one desires economy and comfort.

Many motorists denounce retreading tyes that have become worn, and consequently many owners of small cars do not take advantage of this method of reducing the cost of upkeep of a car. I think, however, it will be found that most of those who decry the retreaded tyre belong to the class of large car owners. It is obvious that a retreaded cover will not last long on the driving wheels of a large car weighing the greater part of two tons; such a car is capable as a rule of great speed, and it would not be wise to fit a worn cover on the front wheels. The case of the small car is very different. Its maximum speed is so low that a very doubtful cover indeed may be run on a front wheel without any fear of disaster arising from a burst. Hence it is always well worth while retreading a cover for use on a small car, even if its condition is rather bad; and if the cover is simply worn the fabric remaining sound and free from damp-rot, retreading is as a rule an exceedingly good investment. Some time ago I removed an 85 mm. cover from the driving wheel of a small car which had been retreaded on one if not two occasions. It was getting in a very bad condition and appeared to me to be past repair, so before throwing it away I thought I would fit it on one of the steering wheels for a little time. I did so, and found that, instead of lasting a few weeks as I imagined, it lasted many months. It never did burst, and it was only the approach of winter that compelled me to remove it.

It is admitted nowadays by everyone that the greater part of the expense of upkeep of a motor car is a question of life of the tyres. Anything that will minimise this expense should be taken advantage of; and in this respect I would advise the owners of small cars not to neglect the prolonged lease of life a cover will obtain at the hands of a first-class firm.

Many medical men who drive their own cars must at times have felt the want of some really good kind of glove that would not only keep the hands warm but at the same time prove impervious to rain and not become icy cold and sodden when driving in wet or snowy weather in winter. For some time I have found the Canadian bag or mitts the most effective hand covering from the point of view of warmth, but have not until recently come across mitts that were unaffected by rain. Recently, however, I have tried a pair of mitts made by Messrs. Samuel Bros., of 65 Ludgate Hill, which are made of horse hide and are consequently waterproof. They are soft and pliable, and have a heavy tufted knit lining with an extra heavy wool-roll top wrist for wind protection. They are the best variety of hand covering I have met with for winter driving, and are not expensive, costing, I believe, 6s. 6d. per pair. "Viator."

